# Chinese English as a Foreign Language Teachers’ Self-Efficacy and Psychological Well-Being as Predictors of Their Work Engagement

**DOI:** 10.3389/fpsyg.2021.788756

**Published:** 2021-11-04

**Authors:** Xiansui Kong

**Affiliations:** School of Foreign Languages, Shandong University of Finance and Economics, Jinan, China

**Keywords:** work engagement, psychological well-being, self-efficacy, English as a foreign language teachers, teaching engagement

## Abstract

Work engagement is widely acknowledged as an influential element in teachers’ professional success; thus, remarkable attention has been paid to the physical and psychological predictors of this construct. Yet, the antecedents of English as a foreign language (EFL) teachers’ work engagement have rarely been studied. To narrow the existing gap, the current inquiry aimed to delve into the psychological determinants of teacher work engagement by scrutinizing the role of psychological well-being and self-efficacy in Chinese EFL teachers’ engagement. To this end, three close-ended questionnaires were given to 304 Chinese EFL teachers *via* WeChat messenger. Conducting correlational analyses, positive and substantial relationships were discovered between psychological well-being, self-efficacy, and teaching engagement. The function that psychological well-being and self-efficacy may serve in Chinese EFL teachers’ engagement was also assessed using path analysis. Chinese EFL teachers’ work engagement was found to be considerably affected by their sense of efficacy and well-being. Some pedagogical implications that might be noteworthy for teachers and institutional administrators are finally discussed.

## Introduction

Teachers in any educational context typically experience a range of difficulties and challenges, yet, most of them are deeply committed to their profession (Greenier et al., 2021). This sense of commitment is technically called “Work Engagement (WE)” which pertains to “a positive, fulfilling and work-related state of mind that is characterized by vigor, dedication, and absorption dimensions” (Schaufeli et al., 2002, p. 75). As put forward by Hakanen et al. (2006), highly engaged teachers devote more energy to fulfilling their professional responsibilities. Bakker et al. (2008) also noted that teachers who demonstrate higher engagement in classroom contexts are more passionate and enthusiastic about their vocation. According to Valenta (2010), teachers’ work engagement can dramatically enhance their effectiveness in academic contexts. He postulated that teachers who are emotionally and physically engaged with their profession commonly put more effort into preparing, designing, and instructing course content, which results in their professional success. Due to the importance of work engagement, over the last two decades, a rising number of studies have been conducted to delve into the determinants of foreign/s language teachers’ engagement (e.g., Saks, 2006; Parker and Martin, 2009; Skaalvik and Skaalvik, 2014; McIlveen and Perera, 2016; Buric and Macuka, 2018; Perera et al., 2018; Chan et al., 2020; Topchyan and Woehler, 2021). Nevertheless, factors contributing to English as a foreign language (EFL) and English as second language (ESL) teachers’ work engagement have rarely been studied (e.g., Greenier et al., 2021; Han and Wang, 2021; Xie, 2021). It implies that the value and significance of this construct in English language classes are not widely recognized by researchers and academics. To address this gap, the current study intends to probe into the role of two important psychological elements, namely, self-efficacy and psychological well-being, on EFL teachers’ work engagement. The value of self-efficacy in promoting teachers’ work engagement is well manifested in “*Social Cognitive Theory* (SCT)” of Bandura (1997) in that self-efficacy is one of the psychological elements that predominate the quantity and quality of human actions. To him, “people often avoid doing tasks which are beyond their capacities, and they do those tasks that they feel they are able to control” (Bandura, 1999, p. 163). Accordingly, self-efficacious teachers who assume that they are adequately capable of instructing learning content, engaging pupils, and managing classroom contexts will put more physical and emotional effort to do so. On the other hand, those teachers who do not believe in themselves and their abilities will demonstrate a low level of engagement in classroom contexts (Bandura, 2001). Besides, the importance of psychological well-being in improving teachers’ work engagement is clearly illustrated by Seligman and Csikszentmihalyi (2000) in their reintroduction of “*Positive Psychology Theory*.” They illustrated that positive emotions, such as “*happiness*,” “*joy*,” and “*satisfaction*,” which are all indicators of one’s psychological well-being, prompt individuals to put more effort into doing activities that they are in charge of (Seligman, 2011). Extending this theory into educational contexts, positive emotions, including contentment, satisfaction, and happiness, that teachers experience in relation to their pupils, colleagues, and working environment result in their psychological well-being (Mercer et al., 2016), which, in turn, improves their work engagement (Dewaele et al., 2019; Wang et al., 2021). Drawing on these theories, examining teachers’ sense of efficacy and psychological well-being in relation to their work engagement seems logical.

Self-efficacy, as a potential predictor of teaching engagement, refers to “peoples’ beliefs about what they can do or how certain they are that they can execute certain actions” (Bong and Skaalvik, 2003, p. 2). Similarly, teachers’ self-efficacy pertains to the degree to which teachers think that they can positively affect their pupils’ academic behaviors and learning outcomes (Friedman and Kass, 2002). This notion was defined by Skaalvik and Skaalvik (2017) as “individual teachers’ beliefs in their own ability to plan, organize, and carry out activities that are required to achieve given educational objectives” (p. 153). As Guidetti et al. (2018) noted, teachers who firmly believe in their professional capabilities are more successful in attaining their instructional goals. Poulou et al. (2019) also mentioned that possessing a sense of self-efficacy empowers teachers to effectively manage classroom contexts.

As another possible determinant of teacher work engagement, psychological well-being generally deals with “one’s degree of happiness and satisfaction with his/her life, work, and physical and mental health” (Garg and Rastogi, 2009, p. 43). Teacher psychological well-being, in particular, pertains to the absence of psychological disorders, including depression, stress, uneasiness, and anxiety (Wong and Zhang, 2014). According to Mercer et al. (2016), teacher psychological well-being serves a pivotal function in the quality of instruction. To them, teachers who enjoy higher psychological well-being can make use of their full potential to teach effectively. Gregersen et al. (2020) also illustrated that psychological well-being supports instructors to teach creatively, use effective instructional strategies, and establish favorable relationships with their pupils.

Given the significance of teachers’ self-efficacy and psychological well-being in educational contexts (Guidetti et al., 2018; Gregersen et al., 2020), a large body of studies have scrutinized the predictors of these constructs (e.g., Klassen and Chiu, 2010; Kurt et al., 2012; Mouton et al., 2013; Bermejo-Toro et al., 2016; Cansoy and Parlar, 2018; Seifalian and Derakhshan, 2018; Fathi et al., 2020; MacIntyre et al., 2020). Likewise, considerable attention has been paid to their educational outcomes (e.g., Huppert, 2009; Klassen and Chiu, 2011; Klassen and Tze, 2014; Helms-Lorenz and Maulana, 2016; Ghasemzadeh et al., 2019; Fathi et al., 2021). Nonetheless, the consequences of these two psychological factors for teachers’ work engagement have remained elusive. That is, a few research studies have focused on the impact of teachers’ sense of efficacy and well-being on their work engagement (e.g., Aiello and Tesi, 2017; Greenier et al., 2021; Han and Wang, 2021). Additionally, among the existing literature, no empirical study in general education or language education has investigated these two constructs simultaneously to assess their power in predicting teacher work engagement. In order to bridge these gaps, the present study sought to delve into the impact of self-efficacy and psychological well-being as determinants of teachers’ engagement in Chinese EFL classes.

## Literature Review

### Teacher Self-Efficacy

The concept of self-efficacy, in a general sense, refers to “individuals’ beliefs about their own capabilities to exercise control over their own level of functioning and over events” (Bandura, 1993, p. 119). When it comes to teaching, self-efficacy deals with the degree to which an instructor believes he/she is capable of leading students toward academic success (Skaalvik and Skaalvik, 2010). As Klassen et al. (2014) noted, self-efficacious instructors are those who trust in their knowledge, competence, and instructional skills. In an endeavor to characterize the nature of teachers’ self-efficacy, Tschannen-Moran and Hoy (2001) divided this construct into three main components of “*efficacy for student engagement*,” “*efficacy for instructional strategies*,” and “*efficacy for classroom management*” (p. 800). Drawing on this categorization, the construct of teacher self-efficacy can be defined as teachers’ evaluation of their capacity to involve pupils in classroom activities, deploy efficient instructional techniques, and manage the learning environment (Lu and Mustafa, 2021). As previous studies revealed, teachers’ self-efficacy is tied with their psychological well-being (Ballantyne and Retell, 2020; Fathi et al., 2020), job satisfaction (Skaalvik and Skaalvik, 2014; Turkoglu et al., 2017; Fathi and Savadi Rostami, 2018), professional commitment (Klassen et al., 2013; Demir, 2020; Nassri and Yaghmaei, 2020), work engagement (Ventura et al., 2015; Buric and Macuka, 2018; Skaalvik and Skaalvik, 2019; Han and Wang, 2021), teaching stress (Fathi and Derakhshan, 2019), and teaching quality (Dimopoulou, 2014; Kunsting et al., 2016; Burić and Kim, 2020).

For one, in a large-scale study, Skaalvik and Skaalvik (2014) probed into the role of teachers’ self-efficacy in their level of work satisfaction. To do this, 2,569 school teachers participated in this study. Participants’ perspectives on the association of self-efficacy with job satisfaction were measured using two valid scales. Drawing on participants’ responses, they found that teachers’ satisfaction at the workplace can positively vary as a function of their self-efficacy beliefs. By the same token, Ventura et al. (2015) studied Spanish EFL teachers’ self-efficacy in relation to their work engagement. To do so, a total of 460 English language teachers willingly took part in this study. Two reliable questionnaires of “*Professional Self-Efficacy* (PSE)” and “*Work Engagement*” were distributed to obtain the needed data. The results of data analysis disclosed a positive association between teachers’ self-efficacy and teaching engagement. In the same vein, Buric and Macuka (2018) scrutinized the role of teachers’ self-efficacy in determining their teaching engagement. To this end, 941 teachers were selected as the participants of the study. Using “*Work Engagement Scale*” and “*Self-Efficacy Scale*,” participants’ attitudes toward the predictive power of self-efficacy were measured. As the results of analyses revealed, participants perceived self-efficacy as a significant determinant of work engagement. Similarly, Han and Wang (2021) examined the association between Chinese teachers’ sense of efficacy and engagement in English language classrooms. To this aim, three reliable questionnaires were administered to 614 EFL teachers who voluntarily took part in the inquiry. The analysis of participants’ viewpoints delineated that teachers’ sense of efficacy is intertwined with their work engagement. Further, in a correlational study, Fathi et al. (2020) explored the association between Iranian English instructors’ self-efficacy beliefs and their psychological well-being. For this, 179 English language instructors were surveyed using two close-ended questionnaires. Analyzing teachers’ responses, the researchers discovered a positive link between teachers’ self-efficacy and their well-being.

### Teacher Psychological Well-Being

The term of psychological well-being is described as one’s appraisal of his or her mental health, pleasure, and satisfaction (Huppert, 2009). In a similar vein, teacher psychological well-being is conceptualized as “individual teachers’ satisfaction with their daily working environment” (Sisask et al., 2014, p. 384). In their study, Dagenais-Desmarais and Savoie (2012) categorized the components of teacher psychological well-being into five dimensions of “*Interpersonal fit at work*,” “*Thriving at work*,” “*Feeling of competency at work*,” “*Perceived recognition at work*,” and “*Desire for involvement at work*.” As Dagenais-Desmarais and Savoie (2012) noted, the first dimension of this construct, namely, “*Interpersonal fit at work*,” is concerned with how teachers perceive their relationships with pupils. “*Thriving at work*” as the second dimension of teachers’ psychological well-being pertains to their perception of doing a meaningful and exciting job. As the third dimension, “*feeling of competency at work*” relates to teachers’ perception of having the essential knowledge and skills to carry out their job responsibilities. “*Perceived recognition at work*,” as the fourth dimension, deals with individual teachers’ impression of being valued for their work. Finally, “*desire for involvement at work*” has something to do with teachers’ willingness to actively engage in educational environments. With regard to the third dimension, the extent to which teachers believe in their abilities is critical for their psychological well-being. That is, self-efficacious teachers will enjoy higher well-being in classrooms contexts (Zee and Koomen, 2016; Jin et al., 2020). As put forward by Brunetto et al. (2012), “teachers who feel well psychologically are more committed to their profession” (p. 430). Ilgan et al. (2015) also mentioned that psychological well-being enables teachers to have a better performance in educational contexts.

To date, some researchers have inspected the impact of psychological well-being on teachers’ organizational commitment (Salimirad and Srimathi, 2016; Jain et al., 2019), job satisfaction (Kurt and Demirbolat, 2019; Hessel et al., 2020), job performance (Çankır and Şahin, 2018; Kumar et al., 2021), and work engagement (Aiello and Tesi, 2017; Greenier et al., 2021). Salimirad and Srimathi (2016), for instance, examined the effects of psychological well-being on teachers’ organizational commitment. To do so, 600 Indian teachers were handpicked from various institutes and schools. In order to collect the required data, participants were given two reliable questionnaires. Inspecting the correlation of questionnaires, the researchers discovered a strong and positive association between teachers’ psychological well-being and organizational commitment. As another instance, Aiello and Tesi (2017) scrutinized the impact of psychological well-being on teacher work engagement. In doing so, 140 Italian teachers were invited to complete two close-ended questionnaires. Analyzing the obtained data, they found a significant interrelationship between teachers’ well-being and engagement. Similarly, Greenier et al. (2021) probed into the role of teachers’ psychological well-being in their work engagement. They attempted to measure the extent to which Iranian and British English language teachers’ work engagement may be affected by their psychological well-being. To this aim, through convenience sampling technique, a total of 363 EFL teachers, including 255 Iranian and 108 British, were selected. Their perceptions regarding the role of teachers’ well-being in their work engagement were gathered using two pre-developed questionnaires. The analysis of obtained data demonstrated that both Iranian and British EFL teachers considered psychological well-being as a positive antecedent of work engagement.

### Teacher Work Engagement

The notion of work engagement is primarily conceptualized as “the state of being emotionally, cognitively, and physically involved in a vocation” (Kahn, 1990, p. 693). This concept was further defined by Maslach and Leiter (1997) as individuals’ perceptions regarding their profession that directly influence their emotional and physical involvement during role performance. Finally, in a more comprehensive definition, Schaufeli et al. (2002) referred to this construct as “a positive, fulfilling and work-related state of mind that is characterized by vigor, dedication and absorption dimensions” (p. 75). In this definition, vigor refers to one’s willingness to devote time and energy to his/her profession. Dedication pertains to a person’s intense passion for his/her vocation, followed by a sense of pride, encouragement, and inspiration. Absorption also refers to a state of being joyfully and deeply absorbed in a particular vocation (Maslach et al., 2008). In light of conception of work engagement of Schaufeli et al. (2002), Cardwell (2011) defined teacher work engagement as “individual teachers’ interest in, enthusiasm for and investment in teaching” (p. 18).

As Klassen et al. (2012) mentioned, engaged teachers who are more focused on, committed to, and enthusiastic about what they actually do in classroom contexts are more successful teachers. It is due to the fact that such teachers typically devote more time and effort to accomplishing their occupational responsibilities (Bakker and Demerouti, 2008). Due to its value, teacher work engagement has been widely studied in the past decades. A group of scholars has focused on the consequences of teacher work engagement (e.g., Hoigaard et al., 2012; Runhaar et al., 2013; Borst et al., 2020). Several researchers have also probed into the antecedents of this construct (e.g., Skaalvik and Skaalvik, 2014; McIlveen and Perera, 2016; Van Der Want et al., 2019; Topchyan and Woehler, 2021). Nonetheless, only a few scholars have investigated the antecedents of ESL/EFL teachers’ work engagement (Greenier et al., 2021; Xie, 2021). To narrow this gap in the literature, the present inquiry aimed to examine the degree to which Chinese EFL teachers’ work engagement may be predicted by their self-efficacy and psychological well-being. Thus, to address the aims of the current study, the following research questions were meticulously formulated as:

1. Are there any significant associations between Chinese EFL teachers’ self-efficacy, psychological well-being, and work engagement?
2. Do Chinese EFL teachers’ self-efficacy and psychological well-being significantly predict their work engagement?

## Materials and Methods

### Participants

Convenience sampling was adopted to select the participants of this inquiry. Convenience sampling, also known as opportunity sampling, is among the most frequently used strategies in which “subjects are typically selected due to their geographical proximity, availability, and easy accessibility” (Dörnyei and Csizér, 2012, p. 81). Following this sampling technique, a total of 304 Chinese EFL teachers who were instructing at 10 different universities in China were included in this study. The sample comprised 160 females (53%) and 144 males (47%), ranging in age from 37 to 58years old. All of them were highly experienced university lecturers whose teaching experience varied from 10 to 25years. Of the total participants, 219 were Ph.D. holders, 59 were Ph.D. candidates, and 26 were MA holders. With regard to academic major, the majority of participants (75%) had studied Applied Linguistics (*N*=137) and Linguistics (*N*=93). The rest (25%) had studied some other English majors, namely, English Language Literature (*N*=32), English Language Translation (*N*=26), and Teaching English to Speakers of Other Languages (*N*=16). To guarantee the trustworthiness of the study, all participants were promised that their demographic information would remain confidential. They also filled out the relevant consent form.

### Instruments

#### Utrecht Work Engagement Scale

To measure participants’ work engagement, “*Utrecht Work Engagement Scale (UWES)*,” developed and validated by Schaufeli et al. (2002), was utilized. This scale comprises three components, including “*Vigour*,” “*Dedication*,” and “*Absorption*.” The UWES consists of 17 items, each of which is scored on a 7-point rating scale. Some instances of UWES’s items are as follows: item (3) “*At my work I always persevere, even when things do not go well*,” item (5) “*At my job, I am very resilient, mentally*,” item (8) “*My job inspires me*,” and item (15) “*It is difficult to detach myself from my job*.” The calculated reliability of UWES in this study was 0.90.

#### Teachers’ Sense of Efficacy Scale

The degree to which Chinese EFL teachers are self-efficacious was assessed *via* a self-report questionnaire, namely, “*Teachers’ Sense of Efficacy Scale (TSES)*” (Tschannen-Moran and Hoy, 2001). The scale includes three underlying components of “*Efficacy for Instructional Strategies*” (items 1–8), “*Efficacy for Classroom Management*” (items 9–16), and “*Efficacy for Student Engagement*” (items 17–24). The TSES is a 5-point Likert scale; the responses to its items can vary from 1 (nothing) to 5 (a great deal). This scale encompasses 24 items, from items included, item (2) “*To what extent can you provide an alternative explanation/example when students are confused*,” item (6) “*How much can you do to adjust your lessons to the proper level for individual students*,” and item (13) “*How well can you keep a few problem students from ruining an entire lesson*.” The TSES enjoyed an acceptable reliability index in the current study (*α*=0.78).

#### Psychological Well-Being at Work

The questionnaire of “*Psychological Well-being at Work (PWBW)*,” designed by Dagenais-Desmarais and Savoie (2012), was employed to measure Chinese EFL teachers’ psychological well-being. The PWBW is comprised of five main dimensions, including “*Interpersonal Fit at Work*,” “*Thriving at Work*,” “*Feeling of Competency at Work*,” “*Perceived Recognition at Work*,” and “*Desire for Involvement at Work*.” This inventory contains 25 items which are rated on a 6-point rating scale (from 0=Disagree to 5=Completely Agree). The following sentences are some examples of PWBW’s items: item (4) “*I feel that my work is recognized*,” item (9) “*I feel that my work efforts are appreciated*,” item (18) “I feel that I know what to do in my job,” and item (22) “*I have a great sense of fulfillment at work*.” The reliability index of PWBW was 0.75 in this inquiry.

### Data Collection

Before commencing the process of data collection, through WeChat messenger, the consent forms were sent to 357 Chinese EFL teachers. Then, the electronic versions of the aforementioned scales (i.e., UWES, TSES, and PWBW) were distributed among those teachers (*N*=304) who were inclined to participate in this study. The respondents were given a thorough explanation of how to fill out the questionnaires. They were also guaranteed that their answers would be treated confidentially and utilized exclusively for the aims of the current inquiry.

### Data Analysis

The datasets were analyzed in four major phases. To begin with, the respondents’ answers were scrutinized to detect and exclude the questionable data. Then, the Kolmogorov-Smirnov test was run to ensure the normality of obtained data. Afterward, through *SPSS (version 28)*, the Pearson correlation coefficient was performed to examine the associations between teachers’ self-efficacy, psychological well-being, and their work engagement. Finally, to examine the role of teachers’ self-efficacy and psychological well-being in their work engagement, path analysis was conducted *via Amos (version 25)*.

## Results

To identify whether the obtained data were distributed normally, the Kolmogorov-Smirnov (K-S) test was applied. The results of K-S test are demonstrated in the table below.

As Table 1 indicates, the Sig values of the normality test (K-S) for teachers’ self-efficacy, psychological well-being, and work engagement are 0.11, 0.20, and 0.09, respectively. With regard to these values, it is plausible to infer that the data were distributed normally. The descriptive statistics of the main constructs, including psychological well-being, self-efficacy, and work engagement, are provided below (Table 2).

**Table 1:** The results of the normality test (K-S).

	Kolmogorov-Smirnov		
	Statistic	df	Sig.
Self-efficacy	0.07	304	0.11
Psychological well-being	0.05	304	0.20
Work engagement	0.06	304	0.09

**Table 2:** Descriptive statistics of the main variables.

	*N*	Min	Max	Mean	SD
Self-efficacy	304	55	178	92.77	10.61
Psychological well-being	304	74	157	106.20	9.48
Work engagement	304	50	162	72.26	8.28

As Table 2 reveals, no outliers or discrepancies were found in the datasets. Table 2 also demonstrated that teacher psychological well-being with a mean score of 106.20, and teacher work engagement with a mean score of 72.26 gained the highest and lowest average points, respectively. The findings of Cronbach alpha analyses for the scales of *Teachers’ Sense of Efficacy*, *Psychological Well-being at Work*, and *Utrecht Work Engagement* are shown hereunder (Table 3).

**Table 3:** The results of Cronbach alpha indices.

Scales	Components	Cronbach alpha
TSES	“Efficacy for Instructional Strategies”	0.73
	“Efficacy for Classroom Management”	0.80
	“Efficacy for Student Engagement”	0.77
	Overall Scale	0.78
PWBW	“Interpersonal Fit at Work”	0.73
	“Thriving at Work”	0.72
	“Feeling of Competency at Work”	0.79
	“Perceived Recognition at Work”	0.71
	“Desire for Involvement at Work”	0.85
	“Overall Scale”	0.75
UWES	“Vigour”	0.82
	“Dedication”	0.75
	“Absorption”	0.76
	Overall Scale	0.90

As shown in Table 3, all of the aforementioned scales attained acceptable Cronbach alpha indices. This implies that the questionnaires used in the current inquiry were highly reliable. To answer the first research question, which deals with the interrelationships of self-efficacy, psychological well-being, and work engagement, Pearson correlation was applied. The following table illustrates the correlation of the variables.

As demonstrated in Table 4, teachers’ self-efficacy was favorably associated with work engagement (*r*=0.33, *n*=304, *p*=0.000, *α*=0.01). Teacher psychological well-being was also positively correlated with work engagement (*r*=0.30, *n*=304, *p*=0.000, *α*=0.01). The Pearson correlation results also delineated a positive connection between teachers’ self-efficacy and psychological well-being (*r*=0.46, *n*=304, *p*=0.000, *α*=0.01).

**Table 4:** Pearson correlation results.

	Self-efficacy	Psychological well-being	Work engagement
***Self-efficacy***			
Pearson correlation	1		
Sig. (two tailed)			
*N*	304		
***Psychological well-being***			
Pearson correlation	0.46	1	
Sig. (two tailed)	0.000		
*N*	304	304	
***Work engagement***			
Pearson correlation	0.33	0.30	1
Sig. (two tailed)	0.000	0.000	
*N*	304	304	304

**Figure 1:**
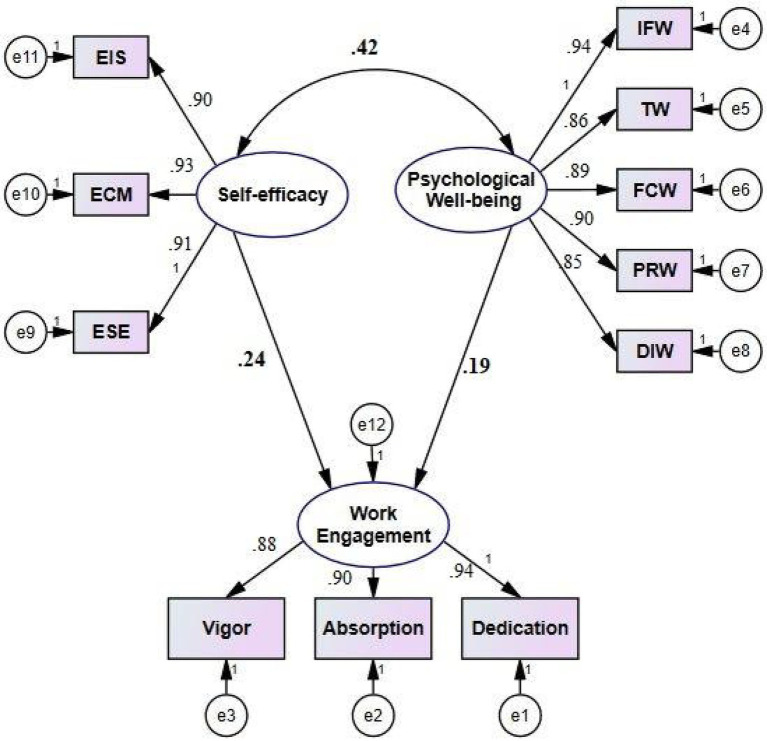
The model of path analysis.

The second research question, which concerned with the predictive power of teachers’ self-efficacy and psychological well-being, was also addressed through path analysis. The results of the path analysis are illuminated in Figure 1.

As diagrammatically illustrated in Figure 1, both self-efficacy (*β*=0.24, *p*<0.05) and psychological well-being (*β*=0.19, *p*<0.05) were found to be strong and positive antecedents of teacher work engagement. The goodness-of-fit indices (i.e., CFI, GFI, NFI, *X*^2^/df, and RMSEA) were used to assess how well the data obtained from participants fit the suggested model.

As illuminated in Table 5, the goodness-of-fit indices were found to be *X*^2^/df=1.85, GFI=0.97, CFI=0.91, NFI=0.96, and RMSEA=0.07, indicating that the suggested model highly fitted the gathered data.

**Table 5:** Goodness-of-fit indices.

	*X*^2^/df	GFI	CFI	NFI	RMSEA
Acceptable fit	<3	>0.90	>0.90	>0.90	<0.08
Proposed model	1.85	0.97	0.91	0.96	0.07

## Discussion

The current inquiry was set to probe into the associations between Chinese EFL teachers’ self-efficacy, psychological well-being, and work engagement. Correlational analyses uncovered a considerable and favorable association, first, between teachers’ self-efficacy and work engagement, and second, between teachers’ psychological well-being and engagement. As to the significant relationship existing between teachers’ self-efficacy and work engagement, it can be noted that this result is consistent with that of Buric and Macuka (2018), who found a direct connection between teachers’ self-efficacy beliefs and teaching engagement. This result is also in line with findings of Skaalvik and Skaalvik (2019), which delineated that teachers’ self-efficacy is closely related to their engagement. The present result also corroborates findings of Han and Wang (2021), indicating that teachers’ self-efficacy is tied with their physical and emotional engagement in classroom contexts. Besides, the discovered association between teacher psychological well-being and work engagement also accords with previous investigations in this area. For one, this finding is consistent with research outcomes of Aiello and Tesi (2017), which depicted that teachers’ well-being in classroom environments is intertwined with their work engagement. This result also lends support to what Greenier et al. (2021) found in their cross-cultural inquiry. They found that English language teachers’ well-being is considerably associated with their active engagement in EFL/ESL classes.

In addition to its primary objective, this study aimed to delve into the predictability of Chinese EFL teachers’ engagement through their self-efficacy and psychological well-being. Put differently, this inquiry sought to determine whether or not Chinese EFL teachers’ engagement is subject to their sense of self-efficacy and psychological well-being. In the model of path analysis, self-efficacy, as an important psychological factor, was shown as a determinant of teaching engagement in Chinese EFL classes. That is, Chinese EFL teachers’ physical and emotional engagement was found to be positively affected by their self-efficacy beliefs. This finding resonates with the result of study of Ventura et al. (2015), illustrating the positive impact of self-efficacy beliefs on teachers’ work engagement. The current finding is also in congruence with the findings of several previous inquiries (e.g., Hakanen et al., 2006; Skaalvik and Skaalvik, 2010; Rogala et al., 2016; Seifalian and Derakhshan, 2018; Ghasemzadeh et al., 2019; Kim and Burić, 2020; Fathi et al., 2021) which identified self-efficacy as the negative predictor of teachers’ disengagement and burnout. The predictability of teachers’ work engagement through their sense of efficacy can be explained by “*Social Cognitive Theory* (SCT)” of Bandura (1997). In SCT, he referred to self-efficacy as one of the psychological factors which are capable of influencing human actions in terms of quantity and quality. He illustrated that “people often avoid doing tasks which are beyond their capacities, and they do those tasks that they feel they are able to control” (Bandura, 1999, p. 163). Thus, it is justified that teachers who have faith in their instructional abilities will be more likely to engage in classrooms.

Besides self-efficacy beliefs, psychological well-being had also a positive impact on Chinese EFL teachers’ work engagement, as delineated by the model of path analysis. This verifies the idea of Wong and Zhang (2014), who postulated that teachers who do not suffer from psychological disorders are more inclined to fulfill their occupational responsibilities. This finding further corroborates what Dagenais-Desmarais and Savoie (2012) stated in this regard. They asserted that teachers’ willingness to actively engage in educational environments is deeply rooted in their psychological well-being. This assertion is consonant with opinion of Brunetto et al. (2012) that “teachers who feel well psychologically are more committed to their profession” (p. 430). The predictability of Chinese EFL teachers’ work engagement through their psychological well-being can also be justified by what Mercer et al. (2016) and Wang et al. (2021) submitted in light of positive psychology theory. They illustrated that positive feelings that teachers experience in their interactions with students, colleagues, and school/university administrators culminate in their psychological well-being, which, in turn, promotes their work engagement. The present finding is in agreement with the findings of previous research studies (Brunetto et al., 2012; Aiello and Tesi, 2017; Greenier et al., 2021), which portrayed that teachers’ engagement in classroom contexts can be considerably improved by their psychological well-being.

## Conclusion

Guided by “Social Cognitive Theory” (Bandura, 1997) and “Positive Psychology Movement” (Seligman and Csikszentmihalyi, 2000), the impact of Chinese EFL teachers’ self-efficacy and psychological well-being on their work engagement was inspected. The findings of correlational and path analyses delineated that self-efficacy and psychological well-being, as two valuable psychological constructs, are highly influential in improving EFL teachers’ work engagement. Hence, it is reasonable to conclude that those English language instructors who enjoy a high degree of psychological well-being and those who firmly believe in their professional abilities and capabilities will demonstrate higher engagement in educational settings. This seems informative and instructive for institutional administrators. To improve teachers’ engagement in classroom contexts, school and university administrators are expected to provide a pleasant environment wherein teachers experience a sense of satisfaction and happiness. Such positive feelings, as Mercer et al. (2016) asserted, will result in teachers’ increased psychological well-being which is critical for their work engagement (Dewaele et al., 2019). The findings of the current inquiry may also be enlightening for those English language teachers who are not inclined to put the effort into executing their occupational tasks. Owing to the fact that a strong belief in personal knowledge and abilities prompts teachers to invest much more time and effort (Buric and Macuka, 2018), they are recommended to improve their sense of efficacy by expanding their instructional knowledge. It is also postulated that it will be insightful if teacher-student interpersonal variables are taken into consideration with respect to teachers’ positive and negative emotions (Xie and Derakhshan, 2021).

Finally, it is worth mentioning that the current study’s results are subject to some important limitations. First and foremost, this investigation was purely quantitative, employing close-ended questionnaires to delve into the participants’ viewpoints. To obtain more reliable outcomes, future inquiries are recommended to use some other instruments, including interviews and open-ended questionnaires, to collect the needed data. Second, in this study, the extent to which situational factors may affect the association of variables was disregarded, which needs to be assessed in future work. Third, this inquiry was conducted in China as an EFL country; hence, the current findings may not be applicable to ESL contexts.

## Data Availability Statement

The original contributions presented in the study are included in the article/supplementary material, and further inquiries can be directed to the corresponding author.

## Ethics Statement

The studies involving human participants were reviewed and approved by the Shandong University of Finance and Economics Research Ethics Committee. The patients/participants provided their written informed consent to participate in this study.

## Author Contributions

XK conceptualized, collected the data, analyzed the data, and drafted the manuscript to submit it to Frontiers in Psychology.

## Conflict of Interest

The author declares that the research was conducted in the absence of any commercial or financial relationships that could be construed as a potential conflict of interest.

## Publisher’s Note

All claims expressed in this article are solely those of the authors and do not necessarily represent those of their affiliated organizations, or those of the publisher, the editors and the reviewers. Any product that may be evaluated in this article, or claim that may be made by its manufacturer, is not guaranteed or endorsed by the publisher.
